# Pre-validation of the WHO organ dysfunction based criteria for identification of maternal near miss

**DOI:** 10.1186/1742-4755-8-22

**Published:** 2011-08-02

**Authors:** José G Cecatti, João P Souza, Antonio F Oliveira Neto, Mary A Parpinelli, Maria H Sousa, Lale Say, Robert C Pattinson

**Affiliations:** 1 Obstetric Unit, Department of Obstetrics and Gynecology, School of Medical Sciences, University of Campinas, Campinas, São Paulo, Brazil; 2 Intensive Care Unit, Department of Obstetrics and Gynecology, School of Medical Sciences, University of Campinas, Campinas, São Paulo, Brazil; 3 Statistics Unit, Center for Studies on Reproductive Health of Campinas (Cemicamp), Campinas, São Paulo, Brazil; 4 WHO Working group on maternal morbidity and mortality, HRP/WHO, Geneva, Switzerland; 5 Department of Reproductive Health and Research, World Health Organization, Geneva, Switzerland

## Abstract

**Background:**

To evaluate the performance of the WHO criteria for defining maternal near miss and identifying deaths among cases of severe maternal morbidity (SMM) admitted for intensive care.

**Method:**

Between October 2002 and September 2007, 673 women with SMM were admitted, and among them 18 died. Variables used for the definition of maternal near miss according to WHO criteria and for the SOFA score were retrospectively evaluated. The identification of at least one of the WHO criteria in women who did not die defined the case as a near miss. Organ failure was evaluated through the maximum SOFA score above 2 for each one of the six components of the score, being considered the gold standard for the diagnosis of maternal near miss. The aggregated score (Total Maximum SOFA score) was calculated using the worst result of the maximum SOFA score. Sensitivity, specificity, positive and negative predictive values of these WHO criteria for predicting maternal death and also for identifying cases of organ failure were estimated.

**Results:**

The WHO criteria identified 194 cases of maternal near miss and all the 18 deaths. The most prevalent criteria among cases of maternal deaths were the use of vasoactive drug and the use of mechanical ventilation (≥1 h). For the prediction of maternal deaths, sensitivity was 100% and specificity 70.4%. These criteria identified 119 of the 120 cases of organ failure by the maximum SOFA score (Sensitivity 99.2%) among 194 case of maternal near miss (61.34%). There was disagreement in 76 cases, one organ failure without any WHO criteria and 75 cases with no failure but with WHO criteria. The Total Maximum SOFA score had a good performance (area under the curve of 0.897) for prediction of cases of maternal near miss according to the WHO criteria.

**Conclusions:**

The WHO criteria for maternal near miss showed to be able to identify all cases of death and almost all cases of organ failure. Therefore they allow evaluation of the severity of the complication and consequently enable clinicians to build a plan of care or to provide an early transfer for appropriate reference centers.

## Background

During the last two decades the reduction in the number of maternal deaths in developed countries and their under estimation in developing countries stimulated the interest for studying and reporting severe maternal morbidity (SMM) or maternal near miss [1-6]. Conceptually, there is a spectrum of clinical severity with two extremes; at one side, the healthy pregnancy, and at the other, the maternal death [7]. The sequence of events or processes that modify the natural evolution of a healthy pregnancy to the maternal death starts after a clinical injury that may be followed by the systemic inflammatory response syndrome (SIRS), going through organ dysfunction and failure, and finally death [8-10].

In this continuum, among the potentially life threatening conditions, there is a specific extreme degree of severe morbidity compatible with the concept of maternal near miss [7,8]. The World Health Organization (WHO) recently defined maternal near miss to describe the condition of a woman that almost died but survived during pregnancy, childbirth or until the 42^nd ^day postpartum [11]. There has been controversy on the concept of maternal near miss [3,12,13], but the discussion has moved on to the current debate on its operational definition. This debate is the consequence of the difficulty in transforming a continuous variable into a discrete one, considering that the best cut off point of the spectrum of severity for its characterization is still not known [14]. A definition based on the recognition of organ dysfunction would be preferable to that based on specific diseases or indicators of management [15].

There are practical difficulties for determining the criteria for a strictly organ dysfunction based definition for international use due to widely varying resources available. There are however tools already validated for recognizing organ dysfunction and failure. The SOFA score, for instance, was extensively validated in general populations for quantifying organ dysfunction [16,17]. For obstetric population however, it is not worldwide recognized as the gold standard for predicting evolution when dysfunctions are present. There are some findings from the population currently under study showing similar results [18].

In order to achieve a situation where the maternal death can be avoided, the cases of SMM and/or maternal near miss must be identified early, feasibly, consistently and uniformly [3,5,19]. Therefore, taking into account the need for a consensus definition and criteria applicable throughout the world, the WHO formed a Working Group on Maternal Mortality and Morbidity Classification. Recently, together with the definition, this group proposed a multi-faceted approach for the criteria and identification of maternal near misses. The criteria include clinical signs, laboratory tests and clinical management. Initially screening should be performed by selecting cases with conditions more commonly associated with severe obstetrical complications, followed by identification of near miss cases by applying the criteria related to organ dysfunction, clinical signs and clinical management from this pre-selected group. The clinical management criteria include special procedures and/or interventions that are not normally necessary during normal pregnancy or postpartum period. Theoretically the set of near miss criteria could be used at any level of health complexity or development [11].

The professionals participating in this WHO Working Group were challenged to test the proposed set of criteria for maternal near miss in their datasets. Therefore the objective of the present study was to retrospectively evaluate the performance of these WHO criteria against markers of organ dysfunction and failure using the maximum SOFA (Sequential Organ Failure Assessment) score [16] as a gold standard. In addition, the study aimed to estimate the indicators of severe maternal morbidity for this population as recently defined by WHO and to appraise the performance of the total maximum SOFA score in predicting maternal near miss cases according to WHO criteria.

## Methods

This study is a pre-validation of the WHO proposed criteria for identifying maternal near miss. It was performed at the Center for Integral Care to Woman's Health (CAISM), in Campinas, Brazil. It is a public university hospital, which is part of the hospital complex from the University of Campinas (UNICAMP), where around 2,900 deliveries take place annually. This facility serves as a tertiary reference center for the regional population of around three million inhabitants and has an intensive care unit conceived specifically for giving appropriate care for women with life threatening conditions during pregnancy and postpartum period.

During the first five years of activity of this obstetric intensive care unit, between October 2002 and September 2007, there were 673 obstetric admissions that were retrospectively analyzed, and among them there were 18 maternal deaths. Women presenting any severe complication related to pregnancy were admitted to the unit as already described elsewhere [20]. All women admitted were considered as having at least one potentially life threatening condition. Among them, were cases of maternal near miss the women with a real life-threatening condition who did not die and fulfilled the criterion for near miss according to the WHO proposal [11]. The data was collected from the clinical records by two researchers plus two research assistants. A protocol for data abstraction was developed, tested and reviewed by the research team before initiation of the study.

Although data were collected for a series of variables related to women and the care they received, for the current approach we used information on the presence of any criterion for maternal near miss as proposed by WHO [11], procedures or interventions related to advanced support to life, indicators of organ dysfunction from the SOFA score [17,21] and the final outcome (maternal death or survivor). The study started only after the approval of the Institutional Review Board. Considering this was a retrospective study evaluating clinical records, informed consent was not required.

The reason for admission to the unit was classified as obstetric if the morbidity was due to a complication directly related to pregnancy or postpartum period, and as clinical-surgical if due to a complication that was previous condition or concomitant to the current pregnancy [22-25]. The maximum SOFA score (0 to 4 points) was used for defining organ dysfunction/failure in cases of SMM [18] and is derived from the original SOFA score [17]. The maximum SOFA score is determined for each one of these six components, using the worst result of each variable across the whole period of admission. For analysis, organ dysfunction was defined as a maximum SOFA score ≥ 1 and ≤ 2, and failure as a score ≥ 3 [16]. The aggregated result is the total maximum SOFA score (TM-SOFA from 0 to 24 points), reflecting the maximum degree of systemic organ dysfunction. The calculation of the score was performed according to the original publication [16,17].

The concept of maternal near miss as proposed by WHO uses criteria based on the identification of clinical signals and symptoms, laboratory tests, and the use of some interventions and/or procedures for its clinical management during any pregnancy or postpartum period [11]. The study was conducted in an environment where high complexity laboratory and technological resources were available, thus variables that can more precisely and uniformly express the systemic organ function in patients were used [16,17,21]. Generally speaking, clinical criteria were not used in this evaluation. The criterion of loss of consciousness lasting ≥ 12 h was not evaluated because there was no consensus definition and the interpretation for cases with continuous sedation was not yet established. There was no registry of uncontrollable seizures, total paralysis and bedside clotting tests in the clinical records evaluated.

Among the eight laboratory criteria proposed by WHO, the oxygen saturation (SAT0_2_) below 90% for 60 minutes or more, and the loss of consciousness and the presence of glucose and ketoacids in the urine, were not routinely registered during the admission of these 673 cases at the unit. The dosage of serum levels of lactate was not included in this analysis because it was also not routinely performed for all cases of SMM admitted. Generally speaking, when performed, its serum measurement was linked with the evaluation of the therapeutic effect following hemodynamic resuscitation in the first 24 h after shock.

### Statistical Analysis

Initially the distribution of all obstetric cases with severe maternal morbidity was evaluated regarding the WHO laboratory and management criteria and according to their outcome i.e. maternal death or maternal near miss, and included estimates of mortality rates per criterion. Afterwards, sensitivity, specificity and positive and negative predictive values of the combined WHO criteria were calculated for the prediction of maternal death and then for organ dysfunction/failure using the maximum SOFA score as a gold standard. The health indicators of maternal morbidity and mortality according to WHO definition were then generated for this specific population according to the recommendation of WHO [11]. Finally, the performance of the total maximum SOFA score was evaluated using its different cut-off points for the prediction of maternal near miss, through its respective ROC curve, now using the combined WHO criteria as gold standard. The area under the curve ROC was used as a measure of the performance [26].

## Results

During the study period, 673 admissions of severe maternal morbidity occurred at the unit, with 18 maternal deaths. The distribution of these cases by WHO criteria according to the outcome (death or near miss) can be observed in Table 1. Among them, 123 women were identified with laboratory criteria and 162 with management criteria. The combination of these criteria (laboratory and management) was able to identify 194 cases of maternal near miss and all the 18 deaths that occurred. The laboratory criteria were present in 15 of 18 deaths (83.3%) and those of management in 17 of 18 deaths (94.4%). The criteria most frequently associated with death were the use of vasoactive drugs and the need of mechanical ventilation (1 h), however the higher mortality rates were for the criteria lower pH, hemodialysis for acute renal failure and cardiopulmonary resuscitation (Table 1).

**Table 1:** Proportion of women dead or surviving (near miss) a severe maternal morbidity according to laboratory, management and combined criteria for maternal near miss proposed by WHO

**WHO criteria**#	Death		Near Miss		Mortality rate (%)
		
	n	%	n	%	
					
**Laboratory based criteria**					
PaO_2_/FiO_2 _<200 mmHg	10	55.6	48	7.3	17.2
Higher creatinine ≥3.5 mg/dl	4	22.2	17	2.6	19.0
Higher bilirubin total >6.0 mg/dl	5	27.8	9	1.4	35.7
Lower pH <7.1	5	27.8	4	0.6	55.6
Lower platelet count <50,000	8	44.4	51	7.8	13.6
					
Any laboratory *	15	83.3	108	16.5	12.2
					
**Management**					
Use of vasoactive drugs	16	88.9	63	9.6	20.2
Hysterectomy	4	22.2	47	7.2	7.8
Blood transfusion ≥5 units	4	22.2	38	5.8	9.5
Invasive mechanical ventilation ≥ 1 h	16	88.9	91	13.9	14.9
Hemodialysis	3	16.7	4	0.6	42.9
CPR	10	55.6	4	0.6	71.4
					
Any management	17	94.4	145	22.1	10.5
					
**Combined (laboratory and management)**	18	100.0	194	29.6	8.5
					
(Total number of SMM)	18		655		

# The clinical criteria were not included in this current evaluation

* The following laboratory criteria were not included: Oxygen saturation <90% for ≥60 min; lactate >5; loss of consciousness with glycosuria and ketonuria

SMM: severe maternal morbidity; CPR: cardio pulmonary resuscitation.

All the 18 maternal deaths occurring at the institution during the period of study were in the intensive care unit (ICU) and all were identified through at least one of the WHO criteria. There were 194 cases presenting life threatening conditions that were afterwards considered as maternal near misses. The remaining 461 cases, although having potentially life threatening conditions, had a good clinical course and did not become near miss cases. Among the women initially admitted to the ICU, the sensitivity of the criteria for identifying maternal deaths was 100%, while the specificity was 70% (Table 2).

**Table 2:** Distribution of women with SMM according to the WHO combined criteria for maternal near miss and outcome of admission to the ICU

Combined WHO criteria for near miss	Outcome of admission *	
	
	Death	Survivor
		
Yes	18	194
No	0	461
		
Total	18	655
		

S = 100.0% [78.1 - 100.0]		Sp = 70.4% [66.7 - 73.8]
		
PPV = 8.5% [5.3 - 13.3]		NPV = 100.0% [99.0 - 100.0]

* Gold standard; [95% CI]

SMM: severe maternal morbidity; ICU: intensive care unit.

**Figure 1:**
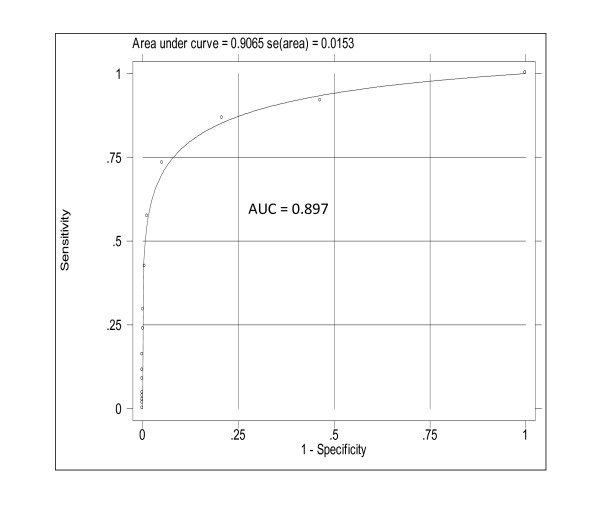
**ROC curve and AUC for total maximum SOFA score with different cut off points as predictor of near misses cases according to the WHO criteria**.

Among the 194 cases of near miss according to WHO criteria, the evaluation of the organ function through the maximum SOFA score identified 120 women with one or more organ failures. From the 76 cases with discordant results between both methods, only one had organ failure and was not identified as maternal near miss by the WHO criteria (Table 3). Figure 1 shows the very good performance of total maximum SOFA score (TM-SOFA) for prediction of maternal near miss cases according to the WHO criteria (AUC = 0.897).

**Table 3:** Distribution of women surviving a SMM according to the WHO combined criteria for near miss and criterion of organ failure in at least one system

Combined WHO criteria for near miss	Criteria of organ failure *	
	
	Yes	No
		
Yes	119	75
No	1	460
		
Total	120	535
		

S = 99.2% [94.8 - 100.0]		Sp = 86.0% [82.7 - 88.8]
		
PPV = 61.3% [54.1 - 68.2]		NPV = 99.8% [98.6 - 100.0]

* Gold standard (SOFA max ≥3 in at least one system) [95%CI]

SMM: severe maternal morbidity.

In the remaining 75 cases with no organ failure, 74 women (98.6%) had one or more management criteria: one criterion in 59 cases, two in 10 cases, three in three cases, and four in two cases. Only one laboratory criterion was alone responsible for the discordance in the unique case (pH < 7.10). The respective values for their sensitivity, specificity, and predictive values are in Table 3.

When considering the cut off point for the identification of organ dysfunction (maximum SOFA score ≥ 1 and ≤ 2) among the 75 discordant cases, 74 were classified as maternal near miss due to management criteria, and 59 women (78.6%) presented one or more organs with dysfunction. Therefore, the combined criteria for organ dysfunction and failure would be able to define 178 women as cases of maternal near miss (91.75%). Among the 16 cases still discordant (without organ dysfunction and/or failure), in 15 women one management criterion was identified (puerperal hysterectomy in eight cases, invasive mechanical ventilation (IMV) in five cases, red blood cell transfusion and use of vasoactive drugs in one case each), and in one case two criteria were identified (puerperal hysterectomy and IMV). The management criteria seem very valuable as they potentially indicate where intervention occurred early enough to prevent organ failure (data not presented in table).

Table 4 shows the indicators regarding severe maternal morbidity as recommended by WHO. The maternal near miss incidence ratio was 13.46 per 1000 LB, while the ratio between maternal near miss and maternal death was 10.7. The high MMR of 124.8 per 100,000 LB is to be interpreted in the context of a tertiary referral hospital.

**Table 4:** Indicators of severe maternal morbidity according to WHO definition

PLTC	MNM	MD	Total	LB
461	194	18	673	14.418
68.5%	28.8%	2.7%		

**MNM incidence ratio**: MNM/LB × 1000 = **13.46/1000 LB**				

**Severe Maternal Outcome Ratio (SMOR) **= (MNM+MD)/LB × 1000				
**SMOR = 14.7/1000 LB**				

**Maternal near miss: mortality ratio**: MNM: 1 MD = **10.7: 1**				

**Mortality index: **MI = MD/(MNM+MD) = **0.085 = 8.5%**				

**Maternal mortality ratio: MMR **= MD/LB × 100.000 = **124.84/100000 LB**				

LB: live births; MD: maternal death; MI: mortality index; MMR: maternal mortality ratio; MNM: maternal near miss; PLTC: potentially life threatening condition; SMOR: severe maternal outcome ratio.

## Discussion

This was the first study to evaluate the performance of laboratory and management criteria proposed by WHO for defining cases of maternal near miss against the organ dysfunction and failure identified by the maximum SOFA score as the gold standard [17,18]. The WHO criteria for maternal near miss performed well and can be used as an effective and valid method for identifying maternal near miss. The information from this study was used by the WHO Working Group on Maternal Mortality and Morbidity Classification, to support the decision of recommending such criteria to be used worldwide [11,27,28].

The prevalence of cases of SMM and/or maternal near miss is variable depending on the criteria used [2,3,13]. In this study, among the 194 cases of maternal near miss defined with criteria proposed by WHO, 119 women (61.34%) had organ failure as defined by the maximum SOFA score. This is in agreement with the current knowledge that the organ dysfunction based criteria constitute the most sophisticated system of maternal morbidity audit [13,19].

In this study, the combined criteria of WHO identified 194 cases of maternal near miss among 673 women with severe maternal morbidity or with potentially life threatening complications. The use of vasoactive drugs and/or mechanical ventilation were more frequent (88.9%) in women who died and this agrees with the findings from other studies showing that the need for hemodynamic and ventilatory support are directly associated with a worse prognosis and higher maternal mortality. This reflects the dysfunction or failure of the cardiovascular and respiratory systems [9,10,18,19,24,29].

The WHO laboratory and management criteria for maternal near miss were able to identify all cases of maternal deaths and had a specificity of 70.4%. In a previous publication, the organ failure of one or more organs was present in 17 of 18 maternal deaths (89.4%), and the total maximum SOFA score ≥ 6 had a sensitivity of 88.9% and specificity of 91.1% [18]. The lower specificity observed by the WHO criteria in predicting outcome was due to the higher number of cases identified as near miss by those criteria (194 women) compared to organ failure (120 women).

When comparing the WHO criteria with the organ failure based ones, we identified 76 discordant cases. There was disagreement in only one case of organ failure without WHO criteria for near miss. This case was a pregnant diabetic woman in hypoglycemic coma after misuse of insulin, which was reverted after a short period of time. At admission she had five points in the Glasgow Coma Scale (maximum neurological SOFA ≥ 3). Considering the criteria used by WHO for evaluation of consciousness is the lowering of its level (Glasgow <10) within a minimum period of time (≥ 12 h), this case, despite the severity and potential for damage, could not therefore be classified as a maternal near miss.

In contrast, 74 women were defined as maternal near miss by management criteria alone, representing 98.6% of discordant cases and 38.1% of all cases of near miss in this study. This result may have been influenced mainly by two ways. First, the cutoff point for defining the laboratory variables of near miss by WHO consists of values compatible with already established organ failure [16,18,20], which increases its specificity, but with a loss of sensitivity. Second, regarding management criteria, where the study was conducted and the available resources should be considered [2]. This study was conducted in a tertiary hospital, where procedures and interventions for monitoring and/or support to life are routinely performed and, in general, without obstacles or resource constraints.

However, as we consider the clinical events or management criteria alone for evaluation, there is a trend to include less severe cases [3]. As an example, a woman with respiratory distress syndrome (RDS) (FIO_2_/PaO_2 _<200 mmHg) and a case with tachypnea (RR > 40 rpm) secondary to pulmonary congestion immediately after delivery, are equally defined as maternal near miss by WHO, however they are in fact different in terms of severity and prognosis. Among the management criteria evaluated, the need for postpartum hysterectomy may be the only one with no parallel in the scores assessing organ function, being present in nine out of the 16 discordant cases of near miss with no organ dysfunction.

The results of the current study showed that the occurrence of maternal near miss in this population using this new definition and criteria was around 13.5 per a thousand live births, an acceptable figure considering the health facility is a tertiary referral university hospital from a middle income country like Brazil, to where the vast majority of real complicated obstetric situations from the whole region are referred to. Some concern may however arise from the fact that SOFA score was used for the first time as the gold standard for identifying organ dysfunction as the ideal way for defining maternal near miss.

The general impression from all the team involved in such initiative was that this classification is relatively easy to use, making it possible to differentiate the most severe cases among all obstetric complications. Therefore the group fully supported the recommendation of its feasibility to be used worldwide, especially considering that this should imply a corresponding appropriate standard of quality care or alternatively a referral to an appropriate health facility with capacity of dealing with that specific clinical situation. However, additional studies should be recommended and welcome, including a specific validation of the clinical criteria, how feasible would be the implementation at primary level through community health care workers, TBA, and a prospective large scale validation of these criteria. This probably would help health systems to better deal with actions towards the reduction of maternal mortality and morbidity as a Millennium Development Goal to be reached soon, and to build a real surveillance system for maternal near miss.

## Conclusions

This study provided an important contribution to the strategies in the management of complications associated with pregnancy, childbirth and postpartum. The first concerns the need for a standard definition of maternal of near miss, already identified, in order to be consistently used and allow comparable results from different contexts. The second refers to the ability of the criteria recently adopted by WHO in predicting maternal deaths and identify the more severe cases who have organ dysfunction or failure. Early recognition of more severe cases is the first step in taking specific measures and, consequently, reduction of maternal mortality, especially in developing settings.

## List of Abbreviations

AUC: area under the curve; ICU: intensive care unit; IMV: invasive mechanical ventilation; LB: live births; MD: maternal death; MMR: maternal mortality ratio; MNM: maternal near miss; PLTC: potentially life threatening condition; RDS: respiratory distress syndrome; SMM: severe maternal morbidity; SOFA: Sequential Organ Failure Assessment; TBA: traditional birth attendant; TM-SOFA: total maximum Sequential Organ Failure Assessment; WHO: World Health Organization.

## Disclosure of interests

The authors declare that they have no competing interests.

## Authors' contributions

JGC and JPS had the original idea for the study. AON, JGC and MAP were responsible for data collection and the plan of analysis. MHS performed the statistical analysis. All authors saw the output of analysis, drew the tables and commented on their significance. AON wrote the first version of the manuscript, then reviewed, amended and corrected by JGC. All authors read the final version of the manuscript and agreed with its content before submission.
